# Conducting a team-based multi-sited focused ethnography in primary care

**DOI:** 10.1186/s12874-017-0422-5

**Published:** 2017-09-12

**Authors:** A.P. Bikker, H. Atherton, H. Brant, T. Porqueddu, J.L. Campbell, A. Gibson, B. McKinstry, C. Salisbury, S. Ziebland

**Affiliations:** 1 Usher Institute of Population Health Sciences and Informatics, The University of Edinburgh, Teviot Place, Dw1, Rm 123, Edinburgh, EH8 9AG UK; 2 Warwick Primary Care, Health Sciences, Warwick Medical School, University of Warwick, Coventry, CV4 7AL UK; 3 Centre for Academic Primary Care, School of Social and Community Medicine, University of Bristol, Canynge Hall, 39 Whatley Road, Bristol, BS8 2PS UK; 4 Nuffield Department of Primary Care Health Sciences, University of Oxford, Radcliffe Primary Care Building, Radcliffe Observatory Quarter, Woodstock Rd, Oxford, OX2 6GG UK; 5 University of Exeter Collaboration for Academic Primary Care (APEx), University of Exeter Medical School, St Luke’s Campus, Smeall Building, Magdalen Road, Exeter, EX1 2LU UK; 6 Department of Health and Social Sciences, University of West England, Glenside Campus, Bristol, BS16 1DD UK; 7 Usher Institute of Population Health Sciences and Informatics, The University of Edinburgh, No. 9 Edinburgh Bioquarter,9 Little France Road, Edinburgh, EH16 4UX UK; 8 Centre for Academic Primary Care, School of Social and Community Medicine, University of Bristol, Canynge Hall, 39 Whatley Road, Bristol, BS8 2PS UK

**Keywords:** Focused ethnography, Research teams, Primary care, Communication technology, Qualitative methods

## Abstract

Focused ethnography is an applied and pragmatic form of ethnography that explores a specific social phenomenon as it occurs in everyday life. Based on the literature a problem-focused research question is formulated before the data collection. The data generation process targets key informants and situations so that relevant results on the pre-defined topic can be obtained within a relatively short time-span. As part of a theory based evaluation of alternative forms of consultation (such as video, phone and email) in primary care we used the focused ethnographic method in a multisite study in general practice across the UK. To date there is a gap in the literature on using focused ethnography in healthcare research.

The aim of the paper is to build on the various methodological approaches in health services research by presenting the challenges and benefits we encountered whilst conducing a focused ethnography in British primary care. Our considerations are clustered under three headings: constructing a shared understanding, dividing the tasks within the team, and the functioning of the focused ethnographers within the broader multi-disciplinary team.

As a result of using this approach we experienced several advantages, like the ability to collect focused data in several settings simultaneously within in a short time-span. Also, the sharing of experiences and interpretations between the researchers contributed to a more holistic understanding of the research topic. However, mechanisms need to be in place to facilitate and synthesise the observations, guide the analysis, and to ensure that all researchers feel engaged. Reflection, trust and flexibility among the team members were crucial to successfully adopt a team focused ethnographic approach. When used for policy focussed applied healthcare research a team-based multi-sited focused ethnography can uncover practices and understandings that would not be apparent through surveys or interviews alone. If conducted with care, it can provide timely findings within the fast moving context of healthcare policy and research.

## Background

The use of ethnographic methods in health services research has slowly grown in popularity although it has been argued that the method is still underused [1–5]. The ethnographic approach can involve multiple data collection methods (e.g. interviews, document reviews), but the approach tends to be defined by the observational fieldwork [5]. This is the immersion of the researcher within a social setting in order to explore the participants’ behaviour and interpretations of the study topic within real time. As such the meaning of the observations is constructed within the interaction of the researcher and participants thereby reflecting the multiple and diverse interpretations of reality [2, 6]. Focused ethnography (FE) is an applied and pragmatic form of ethnography that differs from other ethnographic forms in several ways. Firstly, it explores only one particular problem or topic and has a focused field of enquiry. The background of the problem is studied and based on the literature a problem-focused research question is formulated before going into the field. Secondly, it involves short term and targeted data collection in which the visits to the field are tailored to a particular timeframe or events so that relevant results on the pre-defined topic can be obtained. Lastly, the interviews with carefully selected participants are structured around the study topic [7, 8].

This study used Weiss’ theory based evaluation approach [9] to understand how, under what conditions, for which patients, and in what ways, alternatives to face to face (F2F) consultations such as use of the telephone, email or internet video may offer benefits to patients and practitioners in general practice. A theory based evaluation approach examines the conditions of program implementation and mechanisms which mediate between processes and outcomes as a means to understand when and how programs work [9]. In order to develop the ‘program theory’ we used a realist approach in conducting the focused ethnography. A realist approach acknowledges that the interventions are embedded in multiple social systems and that the context influences the outcome of any intervention. As such, a realist evaluation has an interpretive orientation [10]. Through the ‘implementation theory’ element of the theory based evaluation we then subsequently explored moderating factors which influenced the extent to which the process and outcomes were achieved, such as factors acting as barriers and facilitators to practices offering alternatives to F2F consultations or to different groups of patients using them.

The method of focused ethnography was carefully chosen because it allowed data generation on our pre-defined topic within the context and cultural landscape of the general practices. Health services research, particularly when carried out in primary care setting, often applies a collaborative multisite approach to data collection with collaborators taking responsibility for their ‘site.’ Health services research focuses on team-based research, which is linked to funding bodies that encourage multi-disciplinary team-based research, because of the wider range of expertise that will be brought to the project [8, 10]. Doing a focused ethnography with team members at different sites gives a different dimension to the research process and raises issues such as the impact of the division of tasks on the interpretation of the results. While there has been discussion in the literature about the use of FE (e.g. [7, 8, 11]) and team ethnography (e.g. [12–15]), there is very little published about the conduct of a focused ethnography in a multi-disciplinary team in health services research.

The aim of this paper is to report our experiences, including challenges and solutions when conducting a team-based FE. Our study (the AltCon study) explored the potential of alternatives to face to face consultation in general practice, and the impact on different patient groups.

We use this study as an example to illustrate the benefits and challenges associated with a team FE approach. We use three headings to categorise our experiences: constructing a shared understanding within the team, dividing the tasks within the team, and the functioning of the focused ethnographic team within the broader team of grant holders.

### The AltCon study

Current British healthcare policy and political rhetoric supports the idea that the use of email, telephone and video consultations could enable general practices to provide more convenient, flexible services and there is a drive to put these consultation methods in place as soon as possible [16, 17]. However to date there is little research on whether communication technologies used for consultation are beneficial in primary care, or under which circumstances and for which patient groups they could best be used. The focused ethnography was part of a broader study ‘AltCon’ that aimed to answer the question: under what conditions, for which patients, and in what ways, alternatives to face to face (F2F) consultations may offer benefits to patients and practitioners in general practice? The findings were used to create an on-line resource with recommendations to help individual practices who were interested in implementing alternatives to the F2F consultation.

The overall study was 27 months in duration and consisted of a conceptual review [18], which informed the FE, a survey of general practices on the use of alternatives to face-to-face consultations [19] which aided selection of the case study sites, a quantitative study to explore the feasibility of using routinely acquired data to explore the impact on workload of these alternatives and the FE study itself. The findings of the focused ethnographic study will be published elsewhere [20].

#### Team composition

The team consisted of nine researchers with a range of disciplinary and methodological backgrounds based in four universities in Scotland and England. The team had differing levels of experience in qualitative methodology. The team members’ backgrounds and roles in the study are described in Table 1. The funding was obtained by six primary care researchers (three academic GPs, two social scientists, and one Patient and Public Involvement lead) and some had worked together before. After the funding was obtained three researchers with experience of ethnographic research were employed to conduct the focused ethnographic fieldwork. Although having three researchers collecting data was time efficient, to minimise the costs two of the focused ethnographic researchers were employed from recruitment of the case studies until the in-depth analysis of the collected data (12 months). The third researcher was employed for the duration of the project (27 months) and also took on the role of project manager. This had not been the original proposal – we envisaged a separate project manager role – but feedback from the funders had encouraged us to reduce our costs by combining these two roles in a single post. As such, the structure of the overall team reflected Platt’s [21] observations on funded academic research in which the (three focused ethnographic) study researchers were employed on fixed term contracts to fully focus on the project and the (six) co-investigators were involved in several other studies, had other responsibilities, and more secure positions. We will elaborate later in the paper on the consequences of the team composition on the delivery of the study.

**Table 1:** Background and team member roles in the focused ethnography

Role in project	Task in focused ethnography	Background
1. Co-investigator (SZ)	Senior lead of focused ethnographers and data analysis	Medical Sociologist, background in qualitative research
2. Co-investigator (HA)	Day-to-day lead of focused ethnographers and data analysis	Health services researcher, background in mixed methods, expert in field
3. Project manager (HB)	Focused ethnographic researcher in 3 case study sites	Health psychologist, background in nursing and mixed methods
4. Focused ethnographer (TP)	Focused ethnographic researcher in 3 case study sites	Medical anthropologist, background in qualitative methods
5. Focused ethnographer (AB)	Focused ethnographic researcher in 2 case study sites	Social scientist, background in anthropology and mixed methods
6.Principal investigator (CS)	Data analysis	GP, background in mixed methods, expert in field
7. Co-investigator (BM)	Data analysis	GP, background in mixed methods, expert in field
8. Co-investigator (JC)	Data analysis	GP, background in mixed methods, expert in field
9. Co-investigator (AG)	Data analysis	Patient Public Involvement (PPI) expert

There were three case study site areas and a focused ethnographer was allocated to each area. The focused ethnographic researchers were based in two universities in England and one in Scotland. They had different backgrounds and included medical and social anthropology, nursing and mixed methods research methodologies. This brought a valuable variation in perspective to the research question. Two co-applicants (SZ and HA, see Table 1) managed the three focused ethnographers across the three sites. As such within the team there was a sub-team of five qualitative researchers, the ‘focused ethnographic team’ which we will refer to throughout.

The focused ethnographic case studies took place in eight general practices situated in three geographical areas of the UK; two of the practices were in Scotland, three in Oxfordshire and three in Bristol. The general practices were purposively sampled from the survey findings to represent different experiences of alternative consultation methods and demographics in order to address the predefined research question in different contexts. In line with the focused ethnography method, in each of the eight case studies we used multiple data collection methods that focused only on the participants and interactions that were relevant to the research question. The methods employed in each case study site were non-participant observation of the practice (including the reception, F2F consultations, alternative forms of consultations, practice meetings, waiting room), informal conversations with practice staff, approximately six semi-structured interviews with practice staff who were selected for their knowledge on the topic (this included GPs, nurses, reception staff and the practice manager) and approximately five patients who had experience of either a phone, video or email consultation, and document reviews (i.e. minutes of practice meetings/protocols related to alternative consultation methods). Table 2 shows the eight case study sites, details of the general practices and the period of time spent collecting data in each practice (between 8 and 25 days).

**Table 2:** Description of case study sites and period of observation

Number of registered patients	Location of practice and level of deprivation	Types of alternative to the F2F consultation	No of days spent in observation
18,353	Inner city, Deprived^a^	phone e-consultations, isolated use of email	25
8954	Inner city, Deprived^a^	phone, isolated use of email	19
15,000	Inner city, Mixed^a^	phone, e-consultations & isolated use of email	18
1938	Rural, Mixed^b^	phone, video	8
7196	Inner city, Deprived^b^	phone, e-consultations, isolated use of email	17
13,778	Semi-rural, Affluent^a^	phone, email	25
13,511	Semi-rural, Mixed^a^	phone, email	16
6597	Inner city, Affluent^a^	phone, email	11

^a^Measured by the Index of Multiple Deprivation score

^b^Measured by Percentage of practice patients living in data zones defined as the 15% most deprived (population weighted)

## Reflections on the team-based focussed ethnographic approach

Here we reflect on the methodological lessons learnt. For clarity we have grouped our considerations and experiences into three themes.

### Constructing a shared understanding within the focused ethnographic team

One of the challenges we encountered was how to create and subsequently develop a shared understanding of the phenomenon under investigation within the focused ethnographic team, i.e. the potential impact of the use of alternative consultation methods on primary care practice, staff and patients. One of the recognised aspects of FE is its pre-defined problem focused and conceptual orientation before entering the field. The two focused ethnographic team leads had conducted the conceptual review [18] which was used to inform the focused ethnographic observations, interviews and coding framework. For example, in the review we identified a lack of evidence about the contribution of reception staff to the implementation of alternatives to the face-to-face consultation and thus ensured that they were included in the observations and interviews. The time frame for the project did not allow for the focused ethnographers to be part of this orientation process. Moreover, the three focused ethnographers had different levels of experience in British primary care research and were based in separate geographical locations. For logistical reasons only one focused ethnographer was present at each research site and no other team member visited the site. Because different researchers see and interpret things differently even when observing the same event [14], it was crucial to have mechanisms in place to guide, share and synthesise the fieldwork across the three sites. We did this through drawing on the conceptual review and using it to devise a guidance document for use by the focused ethnographers in the field, face to face workshops, and teleconference calls, as discussed below.

#### Workshops

The focused ethnographic team had three workshops together. The first meeting was before data collection began and had an informal nature. The aim was for the researchers to familiarise themselves with the study and with each other. The leads presented on the background and study materials and on the wider literature. It also included an exercise in which the three ethnographers observed the same setting (i.e. the waiting room at a general practice) so that the team were able to compare styles of working and develop a shared approach. The second and third workshops were more task-oriented and focussed on data sharing, creating coding frameworks for the interview transcripts and field notes, and maintaining team relationships.

#### Standardised documentation

To ensure a cohesive approach to data collection we used standardised documents. Some, such as the participant information leaflets and consent forms had been created for ethical review before the fieldwork, while others such as the case study guide, the interview schedules and coding framework were developed or amended by the FE team during the fieldwork. This flexibility was important to streamline the recording and organising of the field data in such a way that the focused ethnographers worked to the same comprehensible shared format but did not compromise their viewpoint.

The case study guide provided a framework for the researchers to focus on similar elements within the research topic when conducting the fieldwork in the general practices. For example, all researchers would spend a day in the reception of the practice, attend one or more clinics observing a GP conducting face-to-face and alternative forms of consultations, and attend at least one practice meeting and look at any practice protocols on how to use email/phone or video consultations. Throughout the visit there were informal conversations with the staff, as well as audio recorded interviews which were transcribed for analysis. The focused ethnographers recorded their observations and informal conversations in written field notes at the practices and created electronic versions afterwards. These were summarised for sharing amongst the focused ethnographic team.

#### Teleconferences

Regular teleconferences (fortnightly with the focused ethnographic team and weekly between the three focused ethnographers) were held to share emerging observations and issues from the fieldwork. This was to ensure that the study progressed according to plan and any issues arising in the field dealt with often through sharing experiences. For example, during the teleconferences we were able to share tips on recruiting GPs or patients for interviews. The day-to-day lead of the focused ethnographic team led the teleconferences which were preceded by a written update, using a standard format, from each of the ethnographers on their study sites.

The teleconferences were a supportive forum for the focused ethnographers and also functioned as an opportunity to discuss areas of particular interest to the research question that might require further focus. However, as these teleconferences were happening at the same time that the focused ethnographers were “in the field” it was necessary to book these meetings ahead of time with a degree of flexibility to ensure that this did not limit the fieldwork.

#### Field notes

The field notes were a key element of the data collection and central to the data sharing. Inevitably the style of the field notes varied between the researchers from very detailed descriptions of the situations observed, to self-reflections and short notes as can be seen in Table 3.

**Table 3:** Extracts from electronic field notes

Ethnographer 1
I noticed that sometimes the GP used a strange accent which was more obvious when he spoke to some-one from overseas in both face to face consultations and (but less so) telephone consultations! I wondered how much of a researcher effect I was observing
Ethnographer 2
A lady comes in in her 50s saying that she just saw the message on the waiting room screens about the possibility of having telephone consultations and that if she had known before she would have used it. She tried once to speak to the GP for two minutes about her husband’s medication since he was given the wrong prescription and she could not speak to the GP. Her husband had to physically come to speak to the GP about it and she felt that if done over the phone it would have saved him the trouble of coming in. She complained as nobody offered the option to do so. Receptionist tells her that it was the fault of the staff for not offering her the option but that she would still need to book an appointment slot for consultation.
Ethnographer 3
I observe the GP during a telephone clinic. She made 15 calls, asked 5 patients to come to the practice, all came. Calls are arranged in 5 min slots. I observe that she roughly follows the list, though she decides to phone back 2nd patient first. She says that she knows this patient.

From the outset we knew it would be impractical to share the raw field notes. As pointed out by Creese et al. [15] the process of transforming the raw field notes into a format that is workable and acceptable for the team is unique to team-based ethnography. We shared observations through practice summaries, a document with initial codes for each study site. Over the course of the data collection the ethnographers would add new observations to the practice summary template for each of the eight general practices. By doing so the rough field notes (Table 3) were transformed into a standardised format. As a result, comparing the observations between the practices became more straightforward even though the styles between the ethnographers differed. The practice summary provided a trade-off between a systematic approach to identify in the field notes the observations relating to the research question and iteratively developing the initial pre-coding frame further as the fieldwork was conducted. A copy of the practice summary template can be found in Appendix 1.

### Division of tasks between the focused ethnographic team and the wider project team

The focused ethnographic team updated the wider team on the progress of the focused ethnographic case studies approximately every two months either during a conference call or a face-to-face meeting. The meetings were chaired by the chief investigator (CS) and issues relating to the whole project (i.e. the review, survey and patient involvement, as well as associated research opportunities) were also discussed. As such, the chief investigator and co-investigators outside the focused ethnographic team were relatively distanced from the focused ethnographic data collection. At one of the later team meetings, towards the end of the focused ethnographic fieldwork, a discussion was instigated on the challenges this might present for the wider team. The co-investigators were quick to state that they trusted the expertise of the focused ethnographic team, but they were also aware that they had little control or involvement in the data collection or analysis and were looking forward to reassurance that the focused ethnographic team would have useful findings from the field work. The concerns that we discussed are described in Table 4.

**Table 4:** Concerns by role

Focused ethnographers (study researchers)
• Question whether we are doing enough justice to the rich observational data
• Worries related to own relative contribution to the project, am I doing enough?
Leads of ethnographic team (two co-investigators)
• Nature of role is to be involved in several projects at any one time, this can make it difficult
• to stay on top of the data collection process.
• Data management and analysis involves processing large amounts of information and data and the volume can feel overwhelming.
Wider team (chief investigator and three co-investigators)
• Feel a lack of control in project, less ‘hands on’ than in previous work
• Involvement of other projects

Ethnographic teams differ in their structures and divisions of labour [22]. In our case the five-persons focused ethnographic team led the focused ethnographic work with the three focused ethnographers being at the heart of the data collection process as they were the only ones present in each general practice. The focused ethnographers also identified from their interview transcripts, practice summaries and field notes the sections in the data that were relevant to each of the analytical categories from the coding framework that had been agreed within the focused ethnographic team. The focused ethnographers sent the coded data to the day-to-day lead and two additional research assistants were employed to enter the data into NVivo and provide coding reports of the initial analytical categories.

The day-to-day lead of the focused ethnographic team steered the data collection by supervising the focused ethnographers and managing the collected data. Also the day-to-day lead familiarised herself with all the coded interview transcripts and practice summaries to check it for consistency and obtain an insight and overview of the data.

The focused ethnographers experienced tension between on the one hand adhering to the focus on the pre-defined categories and on the other hand the potential loss of explanatory context of the case studies. The day-to-day lead who (like the other co-investigators) was also involved in a number of other research projects felt at times overwhelmed by dealing with the amount of the data that included 80 transcripts (each ranging from 11 to 60 pages) of interviews with patients and primary care staff as well as the practice summaries for each participating practice. In contrast, the CI and several co-investigators were not directly involved with the fieldwork and as such had little control over the data collection process. Not surprisingly, the question arose of how to ensure that the work benefited from and drew more on the skills and knowledge of the overall multi-disciplinary team in order to go beyond comparisons of the sites and draw meaningful conclusions in the analysis phase. An additional challenge was that due to two focused ethnographers being on one year contracts they could not be involved in the analysis of all of the data that they had collected. Future projects would benefit from considering the likely role of each investigator and whether this is practical.

#### Data analysis

Like interviews or focus groups, FE is a data collection method and does not specify the nature of the analysis of the collected data. Following on from the theory based evaluation framework used for the overall project the steps taken to answer the research question were a flexible largely deductive coding and data analysis that were broadly in the realm of the critical realism ontology and epistemology. This involved to agree a thematic coding structure with descriptive labels, use the qualitative software package NVivo to gather related sections of the transcripts and field notes under thematic codes, produce a series of NVivo ‘reports containing all the relevant data across the case study sites and apply the OSOP (one sheet of paper) method [23] to identify the line of argument in each report. Finally we identified outliers or negative cases.

Once the field work was complete and two of the focused ethnographic researchers had completed their posts it was decided that the day-to-day lead, project manager/focused ethnographer and senior lead would read all the coding reports and reflect on the nuances in each code between and within the eight practices. Afterwards they produced condensed summary versions for each code. In order to capitalise on the different disciplines in the wider team the three analysts paired up with one of the clinical co-investigators of the wider team to discuss their reflections on data extracts and summaries. This was the first time that the wider team were involved in helping to interpret the data. The joint analysis was informed by the different insights from a clinical and a social science perspective. The largely deductive approach to data analysis mediated the different qualitative and quantitative methodological backgrounds of the team members. In line with the focused ethnographic and the theory based evaluation approach the analysis excluded the in-depth data on context and other interesting issues that were not within the immediate focus of the pre-defined question. The focused ethnographers whose contracts had ended had agreed to provide further insights if needed. The results and core messages were presented and further discussed at a face-to-face wider team meeting. In addition, we held a stakeholder conference to present and discuss the initial findings and their application. This involved academics, policymakers, healthcare professionals and patients. The discussion at the conference was fed back into the final stages of synthesis of the data.

### Functioning of the team, emotional and practical support

#### Reflexivity and trust in overall team

Trust between the team members has been highlighted as a priority in the literature on team ethnography (e.g. [7, 22]). Based on the exercise by Barry et al. [24] on optimizing teamwork through reflexivity, each member of the wider multi-disciplinary team answered the following two orientating questions at the start of the ethnographic enquiry:

- What will we find is happening in the case study practices?
- What are the main issues that we will encounter in conducting the ethnography?

The answers were circulated by email among the team members for information and discussed at the first workshop of the focused ethnographic team. The personal nature of the exercise set a tone of openness and commitment to the team that continued throughout the project. The three focused ethnographers wrote an additional reflexive account to tease out differences in approach to the field work. They followed the questions in the reflexive tool by Barry et al. [24]: In what way might my experience colour my participation in the project? What experience have I had with qualitative research? What is my orientation to qualitative research? What results do I expect to come out of this project? What theoretical lens do I favour to apply to the results? What is my stake in the research? What do I hope to get out of it? What are my fears? Having reflected on the questions prior to entering the general practice case studies ensured that reflexive and reflective field notes became part of the data collection.

#### Practical support within the ethnographic team

Even though the three focused ethnographic researchers were alone in the field, by focusing on the same pre-defined research question they felt that they were all in a similar situation and felt less isolated as a result. Issues such as difficulties with explaining the nature of focused ethnographic research to the primary care staff, identifying appropriate staff for interviews or dealing with practice staff who did not seem enthusiastic about the research, were to some extent experienced by all. Also, the data collection period for FE is short and intensive. This meant that it was important, though not necessarily easy, to obtain a sense of the practice, and establish rapport with the practice team quickly. Being able to share experiences and discuss strategies to manage certain situations was a unique resource of support and learning opportunity. Concerns were shared and support was given during the teleconferences as well as through emails and phone calls between the telephone meetings. An approachable culture was actively developed from the start and throughout the field work and we felt this was key to the successful delivery of the study.

## Discussion

A team-based FE in multiple general practice sites seemed well suited to the research aim. The approach allowed for observing how those alternative methods of consultations were enacted in situ, in different general practice settings, and contexts, and gathered people’s accounts of their experiences using those methods. Additionally, the team could compare the data within and between the study practices and gain an in-depth understanding of the use of alternative consultations in general practices within the timescale and resources available.

By using our study as an example, this paper reflects on our experiences of conducting a team-based FE in primary care. Other than a guide on conducting focused ethnography [7] there was little guidance about methods for using this approach in primary care with a team in multiple settings. This meant that we had to work things out as we went along. By sharing our experiences we hope to provide a practical insight into this approach for future research.

### Advantages and challenges

There are several advantages to using team-based FE in multiple sites. First, it can be relatively fast as it enables the collection of large amounts of data simultaneously. This means that it is possible to capture insights of concepts and processes within the quickly changing healthcare policy and research context while practices and policies are still relevant, whereas if the field work was completed by one ethnographer over a longer period of time the findings may be reported too late to inform policy decisions. In terms of team working between sites, a benefit was the opportunity for reflection and sense checking to make sure that we stayed on track. Also, working in a team, albeit remotely, made the field work less lonely and isolated for the focused ethnographers.

However, the approach had several challenges. The co-investigators had to try to appoint focused ethnographers who had similar skills, and were likely to get on with each other, something that was challenging when looking for specialist skills across three University cities. Moreover, the data management was a huge task for the day to day lead who oversaw and checked all the coding, and ensured that data were safely and appropriately saved and stored. Research assistants employed in the senior FE lead’s wider team were drafted in to assist with part of the process (there was no funding for this). Another challenge was to ensure the comparability of the varied case studies that were located in very different geographical areas and managed separately by the focused ethnographers, each of whom were bringing their own lens to the data.

### Strengths and limitations

Inevitably, the study was restricted to what was feasible within the available funding resources. Ideally the focused ethnographers would have been able to visit each other’s case research sites in order to obtain a better grasp of how their personal research sites fitted within the overall field. Also, longer contracts for the focused ethnographers would have allowed for more in-depth sharing of the field notes, more face-to-face meetings as well as been able to draw more on the focused ethnographers experiences throughout the data analysis and writing up. The co-investigators from the wider team were less involved in the field work and this was always intended but this meant that the wider team had to rely on the updates of the focused ethnographic team and the lack of involvement caused some concern about whether the study would find anything new. The approachable nature of the team meant that this issue could be discussed. The wider team was engaged at the data analysis stage, which was welcomed by all. Having input from experienced researchers with different disciplinary backgrounds, including general practice, was invaluable to the interpretation and final stages of the project. It meant that we could look at the same data from different angles and tease out the differences in interpretations through discussions. For example, the GPs in the team were in the same professional line of work and worked in a similar setting as the participants that were the focus of the observations. They tended to look more closely at the actions of the observed GPs and nurses (e.g. why a telephone consultation was offered over a face-to-face consultation) and provided background to medical terminology and general practice regulations, while the social scientists focused more on the connections between the events and did not feel the need to justify the actions of the practice staff.

## Conclusions

This project provides an example of how the particular nature of health service research, which is intended to inform the design of health care services, can require the unusual application of established methods. When used for policy focussed applied healthcare research a team-based FE can uncover practices and understandings that would not be apparent through surveys or interviews alone. If conducted with care it can provide timely findings to inform the fast moving developments within the healthcare policy and research world.

## Appendix

**Table 5:** Structured Practice Summary Profile

Practice ID:	What the practice claims to do (from survey, discussions, website etc.	Evidenced by (State who i.e., reception staff, GPs, patients etc.)			
		Observation	Informal Conversations	Formal Interviews	Documentation
**Types** of alternative consultation that are (or were) provided					
**How** these are/were provided (e.g. timing, volume, staffing)					
Parameters for the types of **patients** who are/were encouraged to use these alternatives					

## Abbreviations

AltCon: Alternative Consultation Methods
FE: Focused Ethnography
PPI: Patient Public Involvement
SOP: Standardised Operating Procedure
